# Correlation Between Pre-Operative Sleep Disturbance and Post-Operative Pain in Patients With Rotator Cuff Tear

**DOI:** 10.3389/fnint.2022.942513

**Published:** 2022-06-22

**Authors:** Hui Wu, Wanying Su, Shengtao Huang, Yili Xiao, Liang Lu

**Affiliations:** Hunan Provincial People's Hospital (The First Affiliated Hospital of Hunan Normal University), Changsha, China

**Keywords:** rotator cuff tear, pre-operative sleep disturbance, post-operative pain, arthroscopy, clinical psychology

## Abstract

**Objectives:**

This study aims to investigate the relationship between preoperative sleep disturbance and postoperative pain in patients with a rotator cuff tear, and to provide a theoretical basis for taking corresponding interventions to alleviate postoperative pain in patients with rotator cuff tear.

**Methods:**

A total of 87 patients, who had undergone shoulder arthroscopy due to rotator cuff injury in Hunan Provincial People‘s Hospital from January to October 2021, were selected as the research subjects. The Pittsburgh Sleep Quality Index (PSQI) was used to evaluate the sleep quality of patients with rotator cuff tears. All patients were divided into the low sleep quality group (PSQI score >7 points, *n* = 61) and the high sleep quality group (PSQI score ≤7 points, *n* = 26). Postoperative pain was assessed by using the Numerical Rating Scale (NRS). General clinical data of the patients were collected 1 day, 2 days, and 1 month after surgery. Univariate and multivariate analyses of influencing factors were performed in patients with moderate or above pain at 1 month after surgery.

**Results:**

The score of postoperative pain of patients in the high sleep quality group was significantly lower than that of patients in the low sleep quality group (*P* < 0.05). A total of 35 patients (40.2%) had moderate or above pain 1 month after surgery, including 5 patients (19.2%) in the high sleep quality group and 30 patients (49.2%) in the low sleep quality group. The incidence rate of the low sleep quality group was significantly higher than that of the high sleep quality group (*P* = 0.009). After controlling confounding factors, preoperative sleep disturbance of patients was still independently associated with the occurrence of moderate or above pain 1 month after surgery (OR = 3.794, 95% CI: 1.261–11.409, *P* = 0.018).

**Conclusion:**

Preoperative sleep disturbance can increase the risk of postoperatively moderate or above pain threshold in patients with rotator cuff tear. Paying more attention to and actively improving preoperative sleep disturbance can effectively promote postoperative pain management in patients with rotator cuff tears.

## Introduction

The rotator cuff is composed of tendons of the supraspinatus, infraspinatus, subscapularis, and teres minor muscles, forming a cuff-like structure at the anatomical neck of the humeral head (Smith and Smith, 2010). Rotator cuff injury is one of the most common musculoskeletal diseases with an incidence rate of 16%, which preceded only waist diseases (23%) and knee joint diseases (19%). It is often clinically manifested as shoulder pain and motor dysfunction (Maffulli et al., 2010a,b). Arthroscopic rotator cuff repair is currently one of the main methods for the treatment of rotator cuff injuries (Nicholson et al., 2022), which are often accompanied by different degrees and prolonged postoperative pain (Toma et al., 2019). Poor postoperative pain control will lead to a decrease in the patient's immunity and delay in the recovery of postoperative motor function, resulting in an increased risk of long-term postoperative chronic pain and reducing patient satisfaction (Mulligan et al., 2015). At present, the influence of sleep on pain has aroused widespread concern.

Studies have found that the incidence rate of sleep disturbance in patients with rotator cuff injury is three to six times that of general individuals (Khazzam et al., 2018a; Longo et al., 2019). Although postoperative sleep quality is generally improved after rotator cuff repair, 41% of patients still have postoperative problems and sleep disturbance. Their clinical manifestations are poor sleep quality, insomnia, and frequent awakenings (Uquillas et al., 2016; Horneff et al., 2017). There are many studies related to sleep quality and pain, most of which showed a positive correlation between pain severity and sleep complaints (Lewis, 2016; Longo et al., 2019). However, most of the research subjects have excluded patients with preoperative sleep disturbance. Whether preoperative sleep status affects postoperative pain is unclear. There are few studies targeting on analysis of preoperative sleep disturbance and its correlation with postoperative pain in patients with rotator cuff tear.

Therefore, this study adopted a prospective cohort study to explore the relationship between preoperative sleep disturbance and postoperative pain in patients with rotator cuff injury, to provide targeted nursing interventions, and to improve perioperative pain management for patients with rotator cuff injury and preoperative sleep disturbance.

## Materials and Methods

### Study Subjects

A total of 87 patients who had undergone shoulder arthroscopy due to rotator cuff injury in a Third-class Grade A hospital in Changsha from January to October 2021 were selected as the research subjects. All patients gave informed consent and were approved by the hospital ethics committee. All surgeries were performed by the same surgical team.

#### Inclusion Criteria

(1) Age should be ≥18 years old.
(2) Patients with rotator cuff injury were diagnosed according to symptoms, signs, and imaging examination.
(3) Patients who have no communication barrier.

#### Exclusion Criteria

(1) Patients who refused or could not cooperate with the investigation and signed informed consent.
(2) Patients who had mental disorders, neurological diseases, cognitive disorders, language disorders, or other diseases that affect sleep quality.
(3) Patients with anxiety and depression.
(4) Patients with habitual insomnia, disordered daily schedule, and long-term use of sleep-promoting sedative drugs.
(5) Those with incomplete clinical data.
(6) Those with incomplete postoperative follow-up data.

### Methods

#### Grouping

The Pittsburgh Sleep Quality Index (PSQI) was used to evaluate the sleep quality of patients with rotator cuff injury before surgery. According to the results, patients with rotator cuff injury were divided into the low sleep quality group (PSQI score > 7 points, *n* = 61) and the high sleep quality group (PSQI score ≤ 7 points, *n* = 26). The general information of the two groups of patients and the results of pain and sleep quality assessment at different time points after surgery were collected to understand the effect of preoperative sleep quality on postoperative pain in patients with rotator cuff injury.

#### Research Tool

##### Pittsburgh Sleep Quality Index Scale

PSQI can be used to evaluate sleep disturbance, mental disorders, and sleep quality of general individuals with high reliability and validity, and it is suitable for the evaluation of sleep quality in Chinese patients. PSQI is mainly used to evaluate the sleep quality of the subjects in the last month and consists of 19 self-assessed items and 5 other-assessed items, of which the 19 th self-assessed item and 5 other-assessed items are not involved in the scoring. The remaining 18 self-rating items were divided into 7 dimensions, namely, sleep quality, sleep onset time, sleep time, sleep efficiency, sleep disturbance, use of hypnotic drugs, and daytime dysfunction, respectively. A 4-level scoring method was used, and each dimension was 0–3 points. The cumulative score of each dimension was the total PSQI score, with a total score of 0–21 points. Higher scores indicate poorer sleep quality (Buysse et al., 1989). After the patients were discharged from the hospital, the same dedicated follow-up staff conducted telephone follow-up and completed the sleep evaluation of the PSQI scale, and the PSQI scores of the patients at each time were recorded. The evaluation time was 1 month before and after surgery.

##### Numeric Rating Scale

Pain severity was scored using NRS by 2 investigators. “0” means no pain and “10” means the most severe pain reached unbearable levels. Among them, 1–3 points mean mild pain, indicating that patients can tolerate it; 4–6 points mean moderate pain, indicating that patients suffer from pain and sleep is affected; and 7–9 points mean severe pain, indicating that patients have strong pain, and it is unbearable (Castarlenas et al., 2015). Patients can choose any number from “0 to 10” to express their pain severity by fully feeling their pain. The evaluation time points were 1 day, 2 days, and 1 month before and after surgery, and the pain at 1 month after surgery was evaluated as the average pain score of patients within 1 month.

#### Data Collection

In this study, a questionnaire survey method was conducted by responsible nurses who had received unified training. Before the survey, unified guidelines were used to explain the research object, the meaning, and the method of filling in the questionnaire. Questionnaires were distributed after obtaining the informed consent of patients and their families. The questionnaires were filled out by the research subjects. The investigators answered questions from research subjects by following unified guidelines. For the research subjects who cannot fill it out by themselves, family members or investigators will fill out the form on their behalf by using the inquiry method.

### Statistical Methods

The SPSS 25.0 was used for statistical analysis. Measurement data were expressed as mean ± standard deviation ($\bar{X}$ ± *S*). The *t*-test and Mann-Whitney *U* test were used. Enumeration data were expressed in percent (%) and performed a χ^2^ test. To identify the correlation between a single influencing factor and the moderate or above pain threshold 1 month after surgery, the possible related influencing factors were selected for univariate analysis. To control the confounding factors, the risk factors with *P* < 0.2 in the univariate analysis were included for binary logistic multivariate regression analysis. *P* < 0.05 was considered to be statistically significant.

## Results

### Comparison of General Data of the Two Groups of Patients

The general information, including age, gender, body mass index (BMI), educational level, smoking history, drinking history, etc., of the patients between the high sleep quality group and the low sleep quality group, was not statistically significant (*P* > 0.05) in the comparison of two groups (Table 1).

**Table 1:** Comparison of general data of patients in two groups (*n* = 87).

**Items**		**High sleep quality group (*n =* 26)**	**Low sleep quality group (*n =* 61)**	**Statistical value**	***P-value***
Age ($\bar{X}$ ± *S*, year)		58.20 ± 9.87	59.77 ± 8.68	t = 0.704	0.483
Gender [*n* (%)]	Male	21 (34.4)	9 (34.6)	χ^2^ = 0.000	0.986
	Female	40 (65.6)	17 (65.4)		
BMI/(kg·m^2^)		23.19 ± 2.18	24.28 ± 2.75	T = 1.718	0.089
Educational level [*n* (%)]	Primary education	30 (49.2)	9 (34.6)	Z = −1.036	0.300
	Junior high education	11 (18.0)	6 (23.1)		
	High school and post-secondary education	9 (14.8)	6 (23.1)		
	College degree or above	11 (18.0)	5 (19.2)		
Marital status [*n* (%)]	Married	59 (96.7)	26 (100)	Z = −0.929	0.353
	Single	1 (1.6)	0		
	Widowed	1 (1.6)	0		
Smoking [*n* (%)]	Yes	5 (8.2)	6 (23.1)	χ^2^ = 3.655	0.056
	No	56 (91.8)	20 (76.9)		
Drinking [*n* (%)]	Yes	8 (13.1)	5 (19.2)	χ^2^ = 0.537	0.464
	No	53 (86.9)	21 (80.8)		
Comorbidity [*n* (%)]	Yes	41 (67.2)	16 (61.5)	Z = −0.901	0.367
	No	20 (32.8)	10 (38.5)		

### Pain Scores of Patients in Two Groups at Different Time Points

The pain scores at 1 day, 2 days, and 1 month after surgery in the high sleep quality group were lower than those in the low sleep quality group, and there was a statistical difference between the two groups (*P* < 0.05), as shown in Table 2.

**Table 2:** Pain scores of patients in two groups at different time points after surgery (*n* = 87).

**Items**	**1 day after surgery**	**2 days after surgery**	**1 month after surgery**
High sleep quality group (*n =* 26)	3.269 ± 1.282	2.577 ± 0.987	2.885 ± 1.143
Low sleep quality group (*n =* 61)	4.082 ± 1.646	3.246 ± 1.164	3.426 ± 1.117
*P*	0.028^*^	0.012^*^	0.043^*^

* *P < 0.05*.

### The Sleep Quality and Pain Degree of Patients in Two Groups at 1 Month After Surgery

The sleep quality score in the high sleep quality group was lower than that in the low sleep quality group 1 month after surgery. The number of patients with moderate or above pain 1 month after surgery was less than that in the lower sleep quality group. There was a statistical significance between the two groups (*P* < 0.05), as shown in Table 3.

**Table 3:** Sleep quality and pain degree of patients in two groups at 1 month after surgery (*n* = 87).

**Items**	**Sleep quality**	**Mild pain** **[*n* (%)]**	**Moderate or above pain** **[*n* (%)]**
High sleep quality group (*n =* 26)	5.923 ± 1.623	21 (80.8)	5 (19.2)
Low sleep quality group (*n =* 61)	8.492 ± 2.605	31 (50.8)	30 (49.2)
*P*	0.000^**^	0.009^**^	

** *P < 0.01*.

### Correlation Analysis

The results of univariate analysis of moderate or above pain 1 month after surgery showed that preoperative sleep disturbance was a factor affecting postoperative pain (Table 4). Patients with preoperative sleep disturbance were more likely to experience moderate or above pain threshold after surgery (*P* = 0.009, Table 4). Multivariate analysis showed that after controlling confounding factors, preoperative sleep disturbance was still independently associated with the occurrence of moderate or above pain after surgery (OR = 3.794, 95% CI: 1.261–11.409, *P* = 0.018, as shown in Table 5).

**Table 4:** Univariate analysis of moderate or above pain at 1 month after surgery (*n* = 87).

**Items**		**Mild pain** **(*n =* 52)**	**Moderate or above pain** **(*n =* 35)**	***P-value***
Preoperative sleep disturbance		31	30	0.009^**^
Gender	Male	18	12	0.975
	Female	34	23	
Age (year, $\bar{X}$ ± *S*)		57.924 ± 8.562	59.743 ± 10.799	0.389
BMI (kg·m^2^, $\bar{X}$ ± *S*)		23.742 ± 2.917	23.188 ± 2.421	0.356
Educational level	Primary education	24	15	−1.050
	Junior high education	11	6	
	High school and post-secondary education	12	3	
	College degree or above	5	11	
Marital status	Married	52	33	−1.734
	Single	0	1	
	Widowed	0	1	
Smoking	Yes	7	4	0.780
	No	45	31	
Drinking	Yes	6	7	0.283
	No	46	28	
Comorbidity	Yes	18	12	0.739
	No	34	23	

** *P < 0.01*.

**Table 5:** Logistic regression analysis of moderate or above pain at 1 month after surgery (*n* = 87).

**Influential factors**	**B**	**Wald**	**OR**	**95% CI**	***P*** **value**
Preoperative sleep disturbance	1.333	6.331	3.794	1.261~11.409	0.018^*^
Educational level	0.320	2.532	-	-	0.111
Marital status	19.926	0	-	0.929~2.024	0.999

* *P < 0.05*.

## Discussion

Sleep is considered a dynamic form of rest, allowing reorganization of neuronal activity and energy conservation (Onen et al., 2005). Sleep impacts biological function, physiological recovery, learning, memory, and cognition (Doherty et al., 2019). According to a consensus statement from the American Academy of Sleep Medicine and the Sleep Research Society, sleeping <7 h per night is associated with adverse health outcomes, including weight gain and obesity, diabetes, hypertension, heart disease, stroke, and depression (Watson et al., 2015). Thus, adequate sleep is essential.

Since the main symptoms of rotator cuff injury are shoulder pain and dysfunction, its clinical treatment usually focuses on reducing shoulder pain and restoring the function of the shoulder joint. However, it is easy to ignore that sleep disturbance is also one of the main complaints of patients with rotator cuff injury (Khazzam et al., 2018b; Longo, 2022). Sleep disturbance can exacerbate pain and lead to negative emotions, behavior, and cognition in patients (Woo and Ratnayake, 2020). A previous study (Martin et al., 2018) pointed out that early recognition and effective interventions of preoperative sleep disturbances may reduce postoperative morbidity and health care costs in patients undergoing cardiac surgery. At present, few studies reported the relationship between rotator cuff disease and sleep disturbance, and the correlation between preoperative sleep disturbance and postoperative pain in patients with rotator cuff injury is still unclear.

This study found that among 87 patients with rotator cuff injury, 61 patients had preoperative sleep disturbance (PSQI score > 7 points), accounting for 70.1% of total patients, which was lower than that of Khazzam et al. (2018a) (91%) and Austin et al.' s findings (Austin et al., 2015) (89%). In a systematic review, Kunze et al. (2020) found that the incidence rate of poor preoperative sleep quality in patients with rotator cuff injury was 40.8 to 89%. The results of this study showed that the pain scores of patients in the high sleep quality group were lower than that in the low sleep quality group (*P* < 0.05), and there was a significant difference between these two groups in the incidence rate of moderate or above pain at 1 month after surgery, which is consistent with the study of Sara et al. (2022). After adjusting for confounders, preoperative sleep disturbance remained significant association with moderate or above pain 1 month after surgery.

The reasons may be that postoperative pain in patients with rotator cuff injury is related to sleep disturbance, diabetes, use of external shoulder fixation, and the necessary position at night (Austin et al., 2015; Horneff et al., 2017; Longo et al., 2019). Sleep disturbances may impair important neurophysiological processes, such as interfering with dopaminergic signal transduction, affecting the normal regulation of the endogenous opioid peptide system, and reducing analgesic effects (Finan et al., 2013). There is a two-way relationship between sleep and pain. Pain can delay sleep time, interfere with sleep depth and continuity, and reduce sleep duration and sleep quality. Disturbed sleep quality can increase the body's sensitivity to pain and reduce the body's tolerance to pain, resulting in more severe pain (Andersen et al., 2018; Haack et al., 2020). The results of Fisher et al. (2018) showed that the worse the quality of sleep at night, the higher the incidence rate of pain on the second day, and the worse the pain severity will be Bellomo et al. (2019) found that good preoperative sleep quality has an improvement on experimental pain sensitivity and clinical outcomes for patients after knee arthroplasty. Therefore, improving preoperative sleep disturbance in patients with rotator cuff injury can effectively relieve postoperative pain.

## Conclusion

In this study, the pain scores of patients in the high sleep quality group and low sleep quality group were compared at 1 day, 2 days, and 1 month after surgery. Results indicated that preoperative sleep disturbance can increase the risk of postoperative moderate or above the pain in patients with rotator cuff tear. Our findings suggested that it is necessary to evaluate preoperative sleep disturbance and sleep quality of patients with rotator cuff tear and actively take measures to improve their perioperative pain management.

However, there are some limitations of this study as well. Professional instruments are not enough to monitor the sleep parameters of patients due to the limitation of conditions. Since only the Pittsburgh Sleep Quality Index scale was used to subjectively evaluate the sleep quality of patients, it is difficult to more accurately identify the patients with sleep disorders and analyze the changes in sleep structure. In addition, the total sample size is not sufficient to rule out an impact on the results of the study, which needs a larger sample size and more detailed research to verify the accuracy of this study.

## Data Availability Statement

The raw data supporting the conclusions of this article will be made available by the authors, without undue reservation.

## Ethics Statement

The studies involving human participants were reviewed and approved by Hunan Provincial People's Hospital. The patients/participants provided their written informed consent to participate in this study.

## Author Contributions

HW and WS contributed to the conception and design of the study and wrote the first draft of the manuscript. WS contributed to manuscript revision, reading, and project management. SH, YX, and LL contributed to the data collection and analysis. All authors approved the submitted version.

## Funding

This work was supported by the Health Commission of Hunan Province of China (No. 202114021095). Also the work was supported by Hunan Provincial People's Hospital (The First-Affiliated Hospital of Hunan Normal University).

## Conflict of Interest

The authors declare that the research was conducted in the absence of any commercial or financial relationships that could be construed as a potential conflict of interest.

## Publisher's Note

All claims expressed in this article are solely those of the authors and do not necessarily represent those of their affiliated organizations, or those of the publisher, the editors and the reviewers. Any product that may be evaluated in this article, or claim that may be made by its manufacturer, is not guaranteed or endorsed by the publisher.
